# Metrics for assessing stability of marsh sill living shorelines: Identifying main drivers of marsh boundary degradation

**DOI:** 10.1371/journal.pone.0333214

**Published:** 2025-10-09

**Authors:** Limin Sun, Cindy M. Palinkas, William Nardin

**Affiliations:** Horn Point Laboratory, University of Maryland Center for Environmental Science, Cambridge, Maryland, United States of America; Mykolas Romeris University: Mykolo Romerio Universitetas, LITHUANIA

## Abstract

Marsh sill living shorelines are increasingly common nature-based features in coastal estuaries used to mitigate shoreline erosion and enhance coastal resilience. Evaluating the stability of these structures is crucial for shoreline design and coastal management strategies. Although several metrics have been developed to assess the stability of natural tidal marshes, their suitability for the created marshes of living shorelines is still unclear. This research compiles and analyzes data from 18 marsh sill living shorelines in Maryland, USA — nine with continuous sills and nine with segmented sills. We characterize their eco-geomorphic features and hydrodynamics with 15 metrics through both field sampling and remote sensing. Among the 15 metrics, six representative ones are selected to identify the major factors influencing potential marsh boundary degradation in marsh sill living shorelines. Our findings indicate that living shorelines with ponding at the marsh edge have a significantly higher Unvegetated/Vegetated Ratio (*p* < 0.05) and a lower sediment deposition rate (*p* < 0.1). Gap/Rock ratio and Relative Exposure Index contribute significantly to differences between living shorelines with and without ponding, explaining 5.46% and 4.41% of the variation, respectively (*p* < 0.05 for both). Functional marsh width, introduced here as a novel metric, shows a varying relationship with sediment deposition rate depending on whether the deposition rate exceeded or fell below the relative sea level rise. A marsh width of approximately 5–10 meters appears to optimize both cost-effectiveness and sediment accumulation. By integrating data across regional ecosystems, this study advances our understanding of potential degradation processes in living shorelines, offering valuable insights for shoreline design and post-construction maintenance.

## 1. Introduction

Climate change and coastal urbanization have been accelerating the demand for reducing shoreline erosion and enhancing coastal resilience to storms and sea level rise [1,2]. Traditional hardened structures, like seawalls, revetments, and dikes, have been extensively constructed along coastal shorelines for shore erosion control [3,4]. However, these structures are costly to maintain and can negatively impact intertidal and nearshore habitats [5,6] by disrupting habitat connectivity across the land-water interface [7], increasing water depth [3], causing wave reflection and associated scouring [8], and leading to sediment starvation and loss of intertidal habitat [9,10]. As a result, there is growing interest in identifying and implementing alternative solutions. Natural and nature-based features, also known as nature-based solutions, have emerged as viable options for stabilizing shorelines while preserving coastal ecosystem functions [11]. These features, including dunes, salt marshes, mangroves, seagrasses, rock sills, and oyster reefs, may occur naturally in landscapes or be engineered, constructed and/or restored to mimic natural conditions [12].

Marsh sill living shorelines, consisting of a constructed fringing marsh with adjacent rock sills, are a preferred type of nature-based features for sites with low to moderate erosion rates [13,14]. This approach has been widely implemented in the Chesapeake Bay since the mid-1980s [15]. Sills are typically low-crested structures with a freeboard of approximately 0 to 0.3 m above Mean High Water (MHW), constructed parallel to the shoreline in areas with a small to moderate tidal range, then backfilled with clean sand to achieve a suitable height and slope for planted tidal marsh vegetation [14]. In recent years, marsh sill living shorelines with tidal gaps, also referred to as tidal openings or windows, through the sills have been encouraged to enhance hydrological connectivity [16,17] and allow access for marine fauna (e.g., fish and turtles) to the created marsh [14,15]. While tidal water can access the intertidal marsh behind a continuous sill through pore spaces or via overtopping [15], gaps allow for additional tidal water exchange, wave energy transmission, and sediment transportation between intertidal and subtidal environments.

Previous studies have reported that, although living shorelines are generally effective at wave attenuation and erosion control, their performance can be site-specific [13,18–21]. Site-specific changes can be predicted using multiple linear regression models that consider structure type, shoreline erosion rate, dominant sediment source, and land use [22]. Additionally, the effectiveness of sills diminishes over time due to rising sea levels, gradually reducing the freeboard of the structure [14]. Recent research indicates that accelerated sea-level rise has caused mature marsh sill living shoreline boundaries to convert into open water as ponds expand landward of rock sills, negatively impacting coastal marsh resilience and shoreline stability [23]. Additionally, field observations in segmented sill living shorelines have revealed scouring along the shoreline behind gaps, attributed to waves penetrating this area at the marsh edge [14,24].

Assessing the ecosystem resilience of marsh sill living shorelines is crucial for informing shoreline design and coastal management. Various biotic and abiotic measurements have been developed to evaluate the vulnerability of natural tidal marshes. The success of coastal wetlands restoration is driven by sediment availability [25], and a reduction in the regional marsh area decreases sediment availability in coastal estuarine systems [26]. Sediment deficits and open-water conversion serve as holistic and sensitive indicators of salt marsh vulnerability [27]. The marsh sediment budget (*Q*_b_) provides a spatially integrated measure of competing constructive and destructive forces on sediment dynamics [27]. At landscape scales, *Q*_b_ and sediment-based lifespan (*L*_sed_) scale with the areal unvegetated/vegetated marsh ratios (UVVR), suggesting these metrics are useful indicators of marsh trajectory [27]. Elevation also consistently scales with the UVVR across systems, with lower elevation units demonstrating more open-water conversion and higher UVVRs [28]. However, no single metric universally predicts marsh trajectories, and a more robust approach rigorously combining a suite of spatially integrated, landscape-scale metrics was initially developed [29]. To better integrate these influential factors, Defne et al. [30] established a geospatially resolved wetland vulnerability index (WVI) by ranking values in each dataset to indicate relative vulnerability. Yet, no research has been performed to determine applicable metrics to evaluate ecosystem resilience and predict marsh trajectories of created marshes in marsh sill living shorelines at regional scales.

In this study, we integrate remote sensing and field sampling data from 18 marsh sill living shorelines (9 with continuous sills and 9 with segmented sills) in Maryland’s Chesapeake Bay and Coastal Bays to evaluate marsh stability. We analyzed 15 metrics used to characterize eco-geomorphic features and hydrodynamic conditions of tidal marshes, comparing them between different sill types (continuous versus segmented) and ponding conditions (with ponding versus no ponding). Additionally, we introduced a novel metric, Functional Marsh Width (W*, in m), to describe the vegetation distribution of the narrow fringing marsh band in living shorelines. The primary objectives of this study are: (1) to validate the applicability of potential metrics for assessing the stability of fringing marshes in marsh sill living shorelines, and (2) to investigate the main drivers of potential degradation in marsh sill living shorelines.

## 2. Methods

### 2.1. Study Site

**Fig 1:**
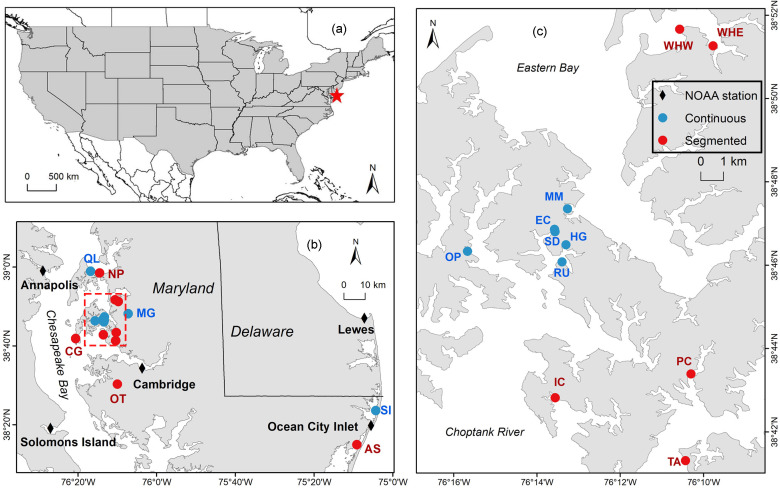
Location of study sites. Black diamonds denote the adjacent NOAA stations. Blue dots indicate the continuous sill living shorelines, and red dots represent the segmented sill living shorelines (see Table 1; Base map from Maryland iMAP (*Maryland Marine Boundaries – Shoreline* dataset, https://data.imap.maryland.gov/datasets/maryland::maryland-marine-boundaries-shoreline/about).

In this study, we analyzed 18 marsh sill living shorelines located in Maryland’s Chesapeake Bay and Coastal Bays (Fig 1). Site selection considered a range of geomorphological characteristics, wave energy exposure, sill configurations, site age, and dominant vegetation type to ensure a representative gradient of environmental conditions relevant to shoreline resilience. All living shorelines included in this study consist of marshes fronted by rock sills installed at the marsh toe, and are located in bays with moderate hydrodynamic conditions. Based on sill construction strategy, the 18 sites were evenly divided into two categories: nine continuous sill living shorelines and nine segmented sill living shorelines (Table 1). Shoreline lengths varied from approximately 50–500 meters, and site ages at the time of sampling ranged from 3 to 15 years. Across all sites, marsh vegetation was dominated by *Spartina alterniflora*, *Spartina patens*, and *Phragmites australis*. Data for Sunset Island (SI) and Assateague State Park (AS) were derived from Sun et al. [23], while data for the remaining continuous and segmented sill living shorelines were sourced from Palinkas et al. [21] and Koontz [31], respectively.

**Table 1:** Name, install year, length, rock sill type, and sampling date for the 18 living shorelines in Maryland.

Site	Install year	Length (m)	Rock sill type	Sampling date	Age during sampling (y)
Sunset Island (SI)	2007	204.2	Continuous	2021-2022	14-15
Queens Landing (QL)	2005	172.9	Continuous	Aug-2018	13
Oppenheim (OP)	2006	134.1	Continuous	Aug-2018	12
Ruesch (RU)	2008	510.5	Continuous	Jul-2018	10
Hatton Garden (HG)	2007	245.5	Continuous	Jul-2018	11
San Domingo (SD)	2007	73.5	Continuous	Jul-2018	11
Environmental Concern (EC)	2005	61.9	Continuous	Oct-2017	12
Myrtle Grove (MG)	2004	246.0	Continuous	Aug-2018	14
Maritime Museum (MM)	2008	53.2	Continuous	Jul-2018	10
Assateague State Park (AS)	2018	212.1	Segmented	2021-2022	3-4
Wye House East (WHE)	2008	330.6	Segmented	Aug-2023	15
Wye House West (WHW)	2009	371.9	Segmented	Sep-2022	13
Tred Avon (TA)	2010	87.6	Segmented	Aug-2022	12
Narrows Pointe (NP)	2016	567.6	Segmented	Sep-2023	7
Plaindealing Creek (PC)	2010	90.3	Segmented	Aug-2022	12
Irish Creek (IC)	2011	141.8	Segmented	Sep-2022	11
Conrad Gordon (CG)	2013	186.4	Segmented	Aug-2023	10
Old Trinity Church (OT)	2008	266.5	Segmented	Sep-2023	15

These sites experience similar tidal ranges and rates of relative sea-level rise. Two sites, SI and AS, were located in the Maryland Coastal Bays, which have a relative sea-level rise rate of 4.94 mm/y and an average tidal range of 0.64 m. The Maryland Coastal Bays is a shallow coastal lagoon system situated between the barrier island of the Delmarva Peninsula and the Atlantic Ocean and is polyhaline [32,33]. The other sites were located in the mesohaline region of the Chesapeake Bay, experiencing relative sea-level rise of ~4 mm/y and tidal ranges of ~0.3–0.5 m [21]. While all of the sites are micro-tidal, significant wind-driven changes in water levels result from variations on the Atlantic continental shelf [34] and from storms, which can flood extensive areas for hours to days [35]. In the mid-bay, most wave action is generated locally and is fetch-limited [36]. While shoreline erosion is the predominant source of sediment in this region, substantial sediment can also be mobilized by overland flow during significant rainfall events [37].

### 2.2. Metrics

This study is based on a variety of data sources, including field sampling, in-situ observations, laboratory processing, and remote sensing. The datasets encompass information on geomorphic features, sediment characteristics, hydrodynamic conditions, vegetation attributes, and marsh trajectory (Table 2).

**Table 2:** Summary of metrics used to assess marsh resilience in living shorelines.

Category	Metric	Definition	Reference or data source
Geographic features	Functional marsh width (W*, m)	Average marsh width along the shoreline	This study
	Gap/Rock (G/R) ratio	Ratio of total gap length to total rock-armored length	[38]
Morphologic features	Elevation relative to Mean Sea Level (m)	Vertical distance of a point above or below Mean Sea Level	USGS National Map 3DEP
	Relative tidal marsh elevation (Z*_MHW_, m)	Normalized marsh elevation within the tidal frame	[39]
Sediment characteristics	Total Sediment Matter (TSM, mg/l)	The concentration of all suspended particles	[40]
	Sand percentage (%)	The proportion of sand-sized particles	[21,23,31]
	Organic matter content (%)	The proportion of organic material	[21,23,31]
Hydrodynamic conditions	Relative Exposure Index (REI)	A measure of how exposed a location is to wind and wave	[41,42]
	Average tidal range (m)	The vertical difference between mean high water and mean low water levels	CO-OPS tide station
	Rate of relative Sea-level Rise (RSLR, mm/y)	The annual change in sea level relative to the land surface	NOAA tide gauge
Vegetation characteristics	Vegetation coverage (%)	The proportion of ground area covered by live vegetation	[21,23,31]
	Stem height (cm)	The vertical measurement of a plant’s stem from the base to tip	[21,23,31]
	Stem density (num/m^2^)	The number of plant stems per square meter	[21,23,31]
Marsh stability	Unvegetated/vegetated ratio (UVVR)	The ratio of the area covered by unvegetated land to the area covered by vegetation	[27]
	Deposition rate (mm/y)	The amount of sediment deposited per year	[21,23,31]

#### Geographic features.

Marsh sill living shorelines are typically characterized by a narrow fringing marsh strip featuring a gradual incline situated behind rock sills, which can be either continuous or segmented in design. At the landward boundary of the marsh, a scarp with higher elevation commonly separates the marsh from the residential area. The seaward boundary with rock sills faces the waves and protects the shorelines from erosion. Recently, degradation of marsh resilience has been observed in some marsh sill living shorelines, with boundaries degraded through open-water conversion as ponds expand landward of the rock sills in both continuous sill [23] and segmented sill living shorelines, suggesting a decline in the stability of marsh sill living shorelines. To assess the impacts of open-water conversion on the trajectory of fringing marshes at living shorelines, we introduce an original metric, Functional Marsh Width (W*, m), to integrate the geographic features of the fringing marshes. The W* is a metric that represents the average width of vegetated marsh along a shoreline. It is calculated as,


$$W^{*}=\frac{A_{v}}{L_{s}}$$

Where *A*_*v*_ is the vegetated marsh area, and the *L*_*s*_ is the total length of the shorelines. S1 Fig of the supporting information illustrates the delineation of vegetated and unvegetated areas at living shorelines with various configurations. This metric provides a standardized measure of marsh extent relative to shoreline length, offering insight into the potential of marshes to buffer wave energy, support habitat, and contribute to shoreline stability.

Hardaway et al. [38] initially defined the Gap/Rock (G/R) ratio for headland breakwaters as the ratio of the gap between breakwater units (*G*_b_) to the breakwater length (*L*_b_). Similarly, we extend this concept to general marsh sill living shorelines. Here, we define the G/R as the ratio of the total length of the gaps between sill segments to the total length of the sill segments. S1 Fig of the supporting information illustrates sill segments and the gaps between them in marsh sill living shorelines with different designs.

#### Morphologic features.

Elevation relative to Mean Sea Level (m) referenced to NAVD88 was derived from 1-meter Digital Elevation Models (DEMs) sourced from the USGS National Map 3DEP (https://www.sciencebase.gov/catalog/item/543e6b86e4b0fd76af69cf4c), and converted to local mean sea level using NOAA’s VDatum software (http://vdatum.noaa.gov/docs/userguide.html). The use of the 3DEP Lidar Base Specification provides standardized and consistent data across collections [43], and NOAA’s VDatum software has been widely employed in previous geomorphic studies [25,27].

Relative tidal marsh elevation (Z*_MHW_, m) is a metric that normalizes elevation to tidal amplitude at mean high water (MHW), it is defined as [39],


$$Z_{MHW}^{*}=\frac{Z-MSL}{MHW-MSL}$$

Where Z is the orthometric elevation referenced to the North American Vertical Datum of 1988 (NAVD88). MSL refers to the mean sea level, and MHW is the mean high water.

Z*_MHW_ is utilized to differentiate wetlands that flood twice a day from those that flood between once a day and a few times a month, accounting for mixed and semi-diurnal tides. This metric provides a convenient and physically relevant means to distinguish between high-elevation and low-elevation marshes. The Z*_MHW_ data used for this study was extracted from a national-scale map with a resolution of 30 × 30 m covering the contiguous USA [39].

#### Sediment characteristics.

Sediment supply is crucial for surface elevation gains in marsh sill living shorelines. To represent local sediment availability to these shorelines, we included the Total Suspended Matter (TSM, mg/l) as a critical metric. TSM in the nearshore coastal water was derived from remotely sensed Moderate Resolution Imaging Spectroradiometer (MODIS) imagery for the Chesapeake Bay and Choptank River (source: NOAA CoastWatch). Satellite-derived TSM measures the concentration of particulate material in surface water, including mud, silt, and other fine-scale debris, encompassing both organic and inorganic fractions [40]. The algorithm links in-water sediment concentration measurements to the amount of light leaving the water that is eventually detected by the satellite [40]. The MODIS-generated TSM product offers high resolution at 250 × 250 m spatial resolution, which is useful for monitoring sediment distribution patterns in the Chesapeake Bay and its tributaries like the Choptank River. In this study, monthly TSM was extracted using Python, averaging the pixel values within 1 km buffers of the study sites. The TSM was then calculated by averaging the TSM values over the 8 months (corresponding to the detection limit for ^7^Be) prior to each site’s field sampling date (refer to Table 1). Specifically, for SI and AS sites, where data encompassed four field sampling periods during 2021–2022, the average value of 24 months’ TSM data extracted from monthly imagery was utilized. Sand percentage (%) and organic matter content (%) were derived from field sampling and subsequent laboratory processing, as detailed in the preceding section.

#### Hydrodynamic conditions.

The Relative Exposure Index (REI) has been extensively applied in previous research to assess wave exposure intensity in coastal zones [21,41,42]. It is defined as


$$REI=\sum_{i=\text{1}}^{\text{8}}\left(V_{i}\times T_{i}\times F_{i}\right)$$

where *i* represents compass headings in 45° increments (e.g., centered on north, northeast, and east). *V*_*i*_ is the average annual wind speed (m/s), *T*_*i*_ is the proportion of time that the wind blew from the *i*-th direction, and *F*_*i*_ is the effective fetch (m) for the i-th direction.

Wind speed data were collected from nearby wind gauges. Specifically, hourly wind directions from Ocean City Inlet, MD (NOAA; ID 8570283) were used for SI and AS. Hourly wind directions from Easton, MD (ID 72404399999) obtained from the National Centers for Environmental Information (NCEI) were used for continuous sill living shorelines at OP, RU, HG, SD, EC, MG, and MM, as well as segmented sill living shorelines at CG, WHE, WHW, PC, IC, and TA. Hourly wind directions from Bay Bridge Airport, MD (ID 72038400124) were obtained for QL and NP. Hourly wind directions from Cambridge, MD (NOAA; ID 8571892) were used for OT.

To calculate effective fetch, the distance from the sampling site to the shoreline along a specified compass bearing [44] was measured for four 11.25° increments centered on the *i*-th compass heading. The effective fetch for each compass heading *i* was then calculated as,


$$F_{i}=\frac{\sum_{j=\text{1}}^{\text{9}}X_{j}\times \text{cos}\;\alpha_{j}}{\sum_{j=\text{1}}^{\text{9}}\text{cos}\;\alpha_{j}}$$

Here, *j* represents the 11.25° increments on either side of and including the compass heading *i*, *X*_*j*_, is the fetch, and *α*_*j*_ is the angle of departure of the *j*-th increment from the compass heading *i* [44].

The tidal range (m) data was obtained and interpolated from nearby National Oceanic and Atmospheric Administration (NOAA) tide stations (https://tidesandcurrents.noaa.gov/). The Rate of relative Sea-level Rise (RSLR, mm/y) information was acquired from the Sea Level Rise Viewer (https://tidesandcurrents.noaa.gov/sltrends/sltrends.html), and was also interpolated using data from NOAA tidal gauges.

#### Vegetation characteristics.

Vegetation data, comprising vegetation coverage (%), stem height (cm), and stem density (num/m^2^), were collected during field sampling. Details about vegetation data collection methods are described in Section 2.3.

#### Marsh trajectory.

The Unvegetated/vegetated ratio (UVVR) is a widely used indicator of tidal marsh trajectory, as it reflects the relationship with the marsh sediment budget [27–30]. Unvegetated areas encompass ponds, channels, and dunes, while vegetated areas are covered by plants within each marsh complex [27]. A stable tidal marsh, with intact marsh plains and minimal deterioration, tends to have a UVVR of around 0.1, and values greater than this indicate a trajectory toward marsh plain deterioration [28]. Previous research on UVVR for tidal marshes at landscape scales used wetland map code delineation [30] or remote sensing imagery, such as the National Agriculture Imagery Program (NAIP [27–29]) and Landsat [45]. Given that marsh sill living shorelines in this study are at a local scale with a maximum length of 570 m, high-resolution images are required to accurately extract marsh boundaries and existing ponding areas. Therefore, this study uses the historical images in 2022 from Google Earth Pro (with spatial resolution up to ~15 cm, depending on location) to delineate vegetated and unvegetated boundaries through manual visualization and digitization. The delineation was conducted by Sun and repeated to assess consistency. Deposition rates (mm/y) were determined using naturally occurring radioisotopes of ^7^Be.

### 2.3. Field and lab methods

Field surveys and sampling were conducted from 2017 to 2023, including vegetation assessments and sediment core collections. Our methodologies for field and laboratory procedures are described in previous studies [21,23,31]. Each site featured three to five transects laid perpendicularly across the intertidal marshes of the living shorelines. Sediment cores were collected, and vegetation parameters were recorded, along each transect at 3–5 points. Data were averaged to provide site-level characterizations.

Briefly, push cores were returned intact to the laboratory and sectioned into 1-cm increments. The top 1–2 cm of each core was analyzed for sand percentage (%), median sediment diameter, organic matter content (%), and ^7^Be-derived sediment deposition rates (mm/y) as described in Palinkas et al. [21]. Vegetation cover (%), stem height, and stem density were recorded by Palinkas et al. [21] and Sun et al. [23].

### 2.4. Statistical analyses

To investigate the differences in metrics between continuous sill living shorelines and segmented sill living shorelines, as well as differences between ponding living shorelines and no ponding living shorelines, we performed a series of t-tests. We then assessed the magnitude of differences between groups by calculating Cohen’s *d* effect sizes [46]. Cohen’s *d* quantifies the standardized difference between group means and provides context for interpreting the practical importance of observed effects. Cohen’s *d* is calculated as:


$$d=\frac{M_{\text{1}}-M_{\text{2}}}{{SD}_{pooled}}$$

where *M*_1_ and *M*_2_ are the means of the two groups, and *SD*_*pooled*_ is the pooled standard deviation.

We interpreted effect sizes following Cohen’s conventional thresholds [46], where values of |d| < 0.2 indicate negligible effects, 0.2 ≤ |d| < 0.5 indicate small effects, 0.5 ≤ |d| < 0.8 indicate medium effects, and |d| ≥ 0.8 indicate large effects. This analysis provides an estimate of the ecological relevance of observed differences, beyond *p*-values alone. Given that our groups (n = 9 for each sill type) are unpaired but balanced, this approach is appropriate and informative for comparing site-level responses across sill types.

Pearson correlation analyses were used to quantify the correlation relationship between pairs of normally distributed metrics, including W*, Z*_MHW_, TSM, Sand percentage, Organic matter content, Average tidal range, Stem height and Deposition rate. For metrics that were not normally distributed, such as G/R ratio, Elevation relative to MSL, REI, RSLR, vegetation coverage, stem density, and UVVR, we used the Spearman coefficient. We conducted the Shapiro-Wilk Test for normality testing. We used a significance threshold of *p* < 0.10, which is more lenient than the conventional *p* < 0.05 commonly used in ecological studies. This choice reflects the limitations of geological field data, which often constrain statistical power [47], and allows for the identification of differences that may still be physically meaningful [48]. We also conducted a series of regression analyses, selecting the best-fitting models based on R^2^, to examine how sediment deposition rate varies in relation to Elevation relative to MSL, Z*_MHW_, TSM, UVVR and W*. All statistical analyses were performed using R.

To assess whether the 18 marsh sill living shorelines grouped by ponding versus no ponding based on the assessed metrics, we conducted a suite of related Non-metric Multidimensional Scaling (NMDS) analyses. Ponding was defined by the relative location of open water to rock sills: if open water was on the marsh side of the delineating line at the end of rock sills, it was classified as a living shoreline with ponding; otherwise, it was categorized as a living shoreline with no ponding. NMDS is a statistical technique used to visualize similarities and dissimilarities of data points, focusing on preserving the order of distances or dissimilarities between points instead of exact distances, which is advantageous for ecological or biological data where measurements can be inherently noisy or are best interpreted in rank order [49]. Although NMDS does not require independence, the interpretation of results and subsequent analyses may be influenced by variable relationships. Therefore, we selected six representative metrics, one from each category, characterizing eco-geomorphic features and hydrological conditions of the living shoreline. The six metrics included G/R ratio, Elevation relative to MSL, sand percentage, REI, stem density, and sediment deposition rate. The metrics were selected for their relative independence and their relevance to both physical and ecological aspects of marsh stability, as well as their ability to capture key functional characteristics of fringing marshes. The Unvegetated/Vegetated Ratio (UVVR) was excluded from multivariate analyses because it described rather than predicted the ponding process. In the analysis, we specified two dimensions and used Bray-Curtis dissimilarity for distance, a commonly preferred method in ecological studies [49]. Differences between ponding and no ponding sites were visualized using a two-dimensional ordination plot highlighting the main contributors. To further analyze differences by ponding and sill type, we created a similarity matrix and conducted an Analysis of Similarity (ANOSIM). We used Similarity Percentages (SIMPER) to examine groupings and identify metrics that best distinguished them as major contributors. SIMPER is a method used in ecological research to determine the contribution of each variable to the dissimilarity between groups, aiding in understanding which variables contribute most to differences observed in multivariate analyses.

## 3. Results

### 3.1. Overall comparisons of living shorelines

The t-test results revealed varying patterns of intergroup differences among the living shorelines categorized by sill configuration (Table 3). In the comparison between continuous and segmented sill types, significant differences were observed primarily in their geomorphic features and vegetation characteristics. Elevations relative to MSL at the segmented sill living shorelines were significantly higher than those at the continuous sill living shorelines with a large effect size (Cohen’s d = –1.22, *p* < 0.05). The Z*_MHW_ showed a similar but slightly less significant pattern between continuous and segmented sill living shorelines (medium effect size, Cohen’s d = –0.74, *p* < 0.1). Regarding vegetation characteristics, significant differences were found in vegetation coverage (large effect size, Cohen’s d = 0.96, *p* < 0.05) and stem height (large effect size, Cohen’s d = 0.91, *p* < 0.1) between continuous and segmented sill living shorelines. Both vegetation coverage and stem height were higher in the continuous sill living shorelines compared to the segmented sill living shorelines. Although stem density was denser in the continuous sill living shorelines compared to the segmented sill living shorelines, this difference was not statistically significant (*p* = 0.3). Importantly, no significant difference was observed in marsh trajectory between continuous sill and segmented sill living shorelines.

**Table 3:** Comparison of metrics for marsh sill living shorelines by sill type (continuous vs. segmented). Values are mean ± standard deviation. T-tests were performed for all comparisons and the effect size is Cohen’s d (n = 9).

Metric	Continuous sill	Segmented sill	*p*-value	Cohen’s d	Magnitude
Functional marsh width (m)	8	9.18	0.5	−0.33	Small
	±0.63	±1.57			
Gap/Rock ratio	0	0.15	**–**	−1.02	Large
		±0.07			
Elevation relative to Mean Sea Level (m)	0.39	0.9	0.02*	−1.22	Large
	±0.11	±0.16			
Relative tidal marsh elevation (Z*_MHW_, m)	2.14	4.83	**0.10.**	−0.74	Medium
	±1.39	±1.15			
Total Sediment Matter (TSM, mg/l)	5.19	5.13	0.9	0.04	Negligible
	±0.20	±0.23			
Sand percentage (%)	64.78	67.66	0.8	−0.14	Negligible
	±7.78	±5.60			
Organic matter content (%)	13.14	12.21	0.8	0.12	Negligible
	±3.03	±2.22			
Relative Exposure Index (REI)	202.35	912.82	0.2	−0.64	Medium
	±27.94	±526.21			
Average tidal range (m)	1.54	1.59	0.7	−0.19	Negligible
	±0.08	±0.08			
Rate of Sea-level Rise (mm/y)	4.7	4.66	0.7	0.17	Negligible
	±0.08	±0.08			
Vegetation coverage (%)	90.5	75.02	0.05*	0.96	Large
	±3.23	±5.67			
Stem height (cm)	125.64	104	**0.07.**	0.91	Large
	±8.58	±7.18			
Stem density (num/m^2)	160.68	114.49	0.3	0.42	Small
	±32.52	±27.36			
Unvegetated/vegetated ratio (UVVR)	0.02	0.11	0.2	−0.73	Medium
	±0.02	±0.05			
Deposition rate (mm/y)	10.74	9.48	0.7	0.33	Small
	±2.90	±1.92			

Notes: *p < 0.05;. *p* < 0.10. Bold values indicate significance.

Differences in metrics between marsh sill living shorelines grouped by ponding types revealed distinct results (Table 4). Living shorelines with ponding tended to exhibit a higher G/R ratio compared to those with no ponding (large effect size, Cohen’s d = −0.94, *p* < 0.1). Although no significant difference was found in elevations between living shorelines with ponding and those with no ponding, there was a significant difference in the average tidal range between the two groups. Sites with ponding had a relatively higher tidal range than those without ponding (large effect size, Cohen’s d = −0.87, **p* *< 0.1). The REI at sites with ponding (1011.05 ± 586.16) appeared higher than at sites with no ponding (194.81 ± 26.32), but this difference was not statistically significant (medium effect size, Cohen’s d = −0.74, *p* < 0.2). Furthermore, the stem height in the living shorelines with ponding was lower than those with no ponding (large effect size, Cohen’s d = 1.25, **p* *< 0.05). In contrast to the comparison between continuous and segmented sill living shorelines, significant differences were observed in marsh trajectory between living shorelines with ponding and those with no ponding. Specifically, the UVVR of the living shorelines with ponding (0.14 ± 0.06) was notably higher than those with no ponding (0, large effect size, Cohen’s d = −1.39, *p* < 0.05), while the sediment deposition rate at sites with ponding (7.14 ± 1.50 mm/y) was relatively lower than those with no ponding (11.59 ± 2.49 mm/y, medium effect size, Cohen’s d = 0.68, *p* < 0.1).

**Table 4:** Comparison of metrics for marsh sill living shorelines by ponding condition (no ponding vs. ponding). Values are mean ± standard deviation. T-tests were performed for all comparisons and the effect size is Cohen’s d (n = 9).

Metric	No ponding	Ponding	*p*-value	Cohen’s d	Magnitude
Functional marsh width (m)	8.07	9.24	0.50	−0.33	Small
	±0.60	±1.76			
Gap/Rock ratio	0.01	0.15	**0.10.**	−0.94	Large
	±0.01	±0.08			
Elevation relative to Mean Sea Level (m)	0.51	0.81	0.20	−0.64	Medium
	±0.16	±0.13			
Relative tidal marsh elevation (Z*_MHW_, m)	3.38	3.61	0.90	−0.06	Negligible
	±1.39	±1.13			
Total Sediment Matter (TSM, mg/l)	5.07	5.22	0.60	−0.24	Small
	±0.21	±0.19			
Sand percentage (%)	62.48	70.89	0.40	−0.42	Small
	±6.27	±7.08			
Organic matter content (%)	14.21	10.75	0.40	0.45	Small
	±2.42	±2.80			
Relative Exposure Index (REI)	194.81	1011.05	0.20	−0.74	Medium
	±26.32	±586.16			
Average tidal range (m)	1.48	1.67	**0.10.**	−0.87	Large
	±0.02	±0.12			
Rate of Sea-level Rise (mm/y)	4.69	4.67	0.90	0.08	Negligible
	±0.07	±0.09			
Vegetation coverage (%)	84.60	84.78	0.90	−0.01	Negligible
	±5.44	±5.18			
Stem height (cm)	127.08	99.50	0.02*	1.25	Large
	±6.12	±8.83			
Stem density (num/m^2)	143.72	139.27	0.90	0.05	Negligible
	±32.96	±25.65			
Unvegetated/vegetated ratio (UVVR)	0	0.14	0.03*	−1.39	Large
		±0.06			
Deposition rate (mm/y)	11.59	7.14	**0.10.**	0.68	Medium
	±2.49	±1.50			

Notes: *p < 0.05;. *p* < 0.10. Bold values indicate significance.

### 3.2. Correlation matrix for the metrics

The cross-correlation analysis of the metrics (Table 5) revealed several significant relationships. The largest correlation was observed between the average tidal range and stem height (*r* = −0.740). Additionally, the average tidal range showed significant correlations with W* (*r* = 0.527) and sediment deposition rate (*r* = −0.382), indicating the influence of tidal conditions on marsh trajectory in living shorelines. Moreover, the sediment deposition rate exhibited inverse correlations with the UVVR (*r* = −0.393), another critical indicator for marsh trajectory. The sediment deposition rate was positively correlated with Z*_MHW_ (*r* = 0.424) and TSM (*r* = 0.579). Notably, the UVVR was positively correlated with the G/R ratio (*r* = 0.569) and negatively correlated with stem height (*r* = −0.583). However, no significant correlation was found between UVVR and any relative elevation metrics (elevation relative to MSL or Z*_MHW_), suggesting that ponding processes in marsh sill living shorelines may differ from those in natural marshes. The remaining variables generally showed no significant correlation with marsh trajectory based on these analyses. These findings provide insights into the key factors influencing marsh resilience and trajectory in marsh sill living shorelines.

**Table 5:** Correlation coefficients. Red indicates significantly positive correlations. Blue shows significantly negative correlations.

	W*	G/R	*E*_MSL_	Z*_MHW_	TSM	SP	OMC	REI	ATR	RSLR	VC	SH	SD	UVVR	DR
W*	1														
Gap/Rock ratio	0.294	1													
Elevation to MSL	0.271	**0.610**	1												
Z*_MHW_	−0.210	0.303	**0.416**	1											
TSM	−0.168	0.118	0.379	0.254	1										
Sand Percentage	**0.464**	0.264	0.352	0.002	0.288	1									
Organic Matter Content	−0.114	−0.186	−0.389	−0.084	−0.368	−0.798	1								
REI	0.110	0.354	0.300	0.226	0.344	0.311	**−0.655**	1							
Average Tidal Range	**0.527**	0.268	0.105	−0.188	−0.184	0.334	−0.006	−0.380	1						
Rate of Sea-level Rise	0.055	−0.092	−0.215	−0.293	−0.075	0.304	−0.299	−0.073	0.055	1					
Vegetation Coverage	0.203	**−0.435**	−0.385	−0.074	−0.188	−0.032	−0.109	0.241	−0.218	−0.319	1				
Stem Height	**−0.554**	**−0.614**	−0.375	0.109	0.163	**−0.422**	0.309	−0.327	**−0.740**	−0.278	0.068	1			
Stem Density	0.120	−0.109	−0.037	−0.145	0.181	0.167	0.064	0.010	−0.222	**−0.405**	0.407	0.152	1		
UVVR	0.061	**0.569**	0.274	−0.029	0.048	0.230	−0.247	0.349	0.396	0.018	−0.237	**−0.583**	−0.055	1	
Deposition Rate	−0.191	0.008	0.307	**0.424**	**0.579**	−0.041	−0.005	0.040	**−0.382**	−0.221	−0.191	0.321	0.314	**−0.393**	1

### 3.3. Metrics related to marsh trajectory of living shorelines

**Fig 2:**
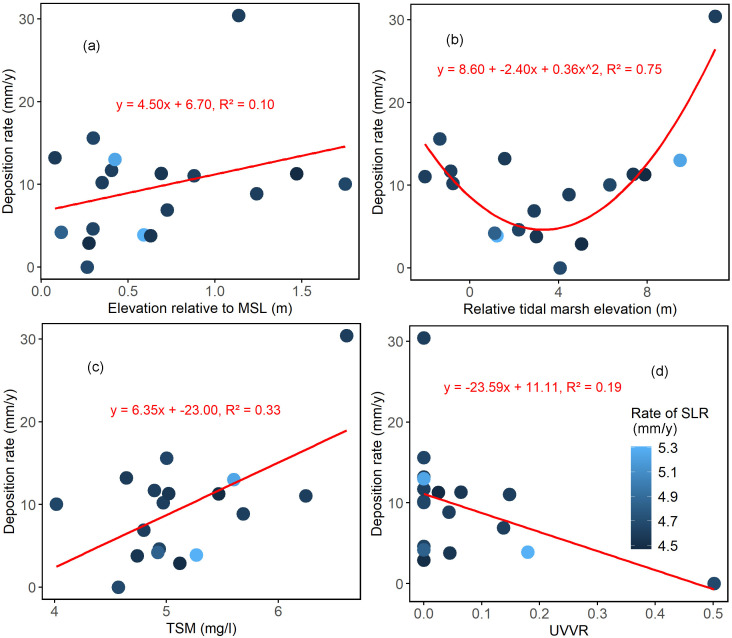
Sediment deposition rate (mm/y) in relation to (a) Elevation relative to Mean Sea Level (MSL, m), (b) Relative Tidal Marsh Elevation (Z*_MHW,_ m), (c) Total Suspended Matter (TSM, mg/L), and (d) Unvegetated/Vegetated Ratio (UVVR). Different colors correspond to different rates of sea level rise (mm/y).

The results revealed several notable relationships between the critical metrics and sediment deposition rate (Fig 2). Firstly, a weak positive linear relationship was observed between elevation relative to MSL and sediment deposition rate (Fig 2a). Secondly, a strong polynomial relationship was found between Z*_MHW_ and sediment deposition rate (R^2^ = 0.75; Fig 2b), likely linked to the significant correlation observed between average tidal range and deposition rate (Table 4). Additionally, a significant positive relationship (R^2^ = 0.33) was found between TSM and sediment deposition rate, indicating higher sediment deposition in marshes located in living shorelines with greater sediment availability in the nearshore coastal water (Fig 2c). Moreover, a negative relationship (R^2^ = 0.19; Fig 2d) was observed between the UVVR and sediment deposition rate, both of which are indicators used to assess marsh trajectory. Sites with a UVVR of 0, indicating no ponding, were observed at several locations. Specifically, QL exhibited a particularly high sediment deposition rate, appearing as an outlier in this correlation (Fig 2d). After removing QL from the analysis, the correlation between UVVR and deposition rate improved (S2a Fig; y = −18.52x + 9.63, R^2^ = 0.28). Further filtering out data points with a UVVR of 0 enhanced the correlation even more (S2b Fig; y = −19.57x + 9.94; R^2^ = 0.52), highlighting the role of ponding in influencing sediment deposition and marsh trajectory in these living shorelines. These findings provide valuable insights into the complex relationships between physical and ecological factors impacting marsh resilience and trajectory in marsh sill living shorelines.

**Fig 3:**
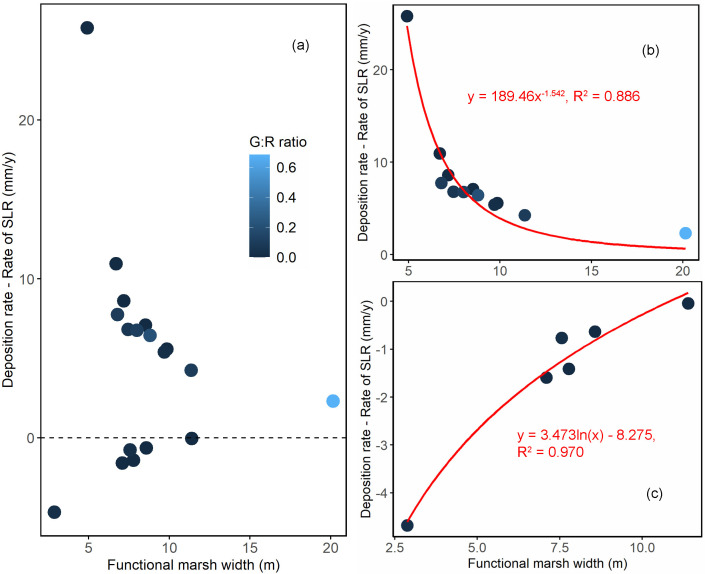
Relationship between functional marsh width (W*, m) and the difference between the sediment deposition rate and the Rate of relative Sea-Level Rise (RSLR; mm/y). **(a)** Scatter plot illustrating the relationship between W* and the difference between the sediment deposition rate and RSLR. **(b)** Relationship between W* and the difference between the sediment deposition rate and RSLR when the sediment deposition rate exceeds the RSLR. **(c)** Relationship between W* and the difference between the sediment deposition rate and RSLR when the sediment deposition rate is below the RSLR. Different colors represent varying Gap/Rock ratios.

Although no linear relationship was identified between the W* and deposition rate, we found nonlinear relationships between W* and the difference between the observed sediment deposition rate and the rate of relative sea-level rise (Fig 3). The results depicted varying patterns in the scaling of sediment deposition rate with W* under different circumstances. When the deposition rate exceeded the rate of sea level rise, a negative scaling relationship was observed (Fig 3b), indicating that as the functional marsh width increased, the deposition rate also increased (y = 189.46x^-1.542^, R^2^ = 0.886). Conversely, when the deposition rate was below the rate of sea level rise, a positive scaling relationship was evident (Fig 3c), signifying that the deposition rate increased with increasing functional marsh width (y = 3.473ln(x) – 8.275, R^2^ = 0.970). In general, a width of 5–10 m would represent a good balance between cost-effectiveness and achieving a relatively high deposition rate under both circumstances described. This functional marsh width optimizes sediment deposition rates, showing favorable outcomes regardless of whether the deposition rate exceeds or underperforms the rate of sea level rise. A summary of the regression models for sediment deposition rate is provided in S1 Table of the Supporting Information.

### 3.4. Factors potentially related to ponding processes in living shorelines

**Fig 4:**
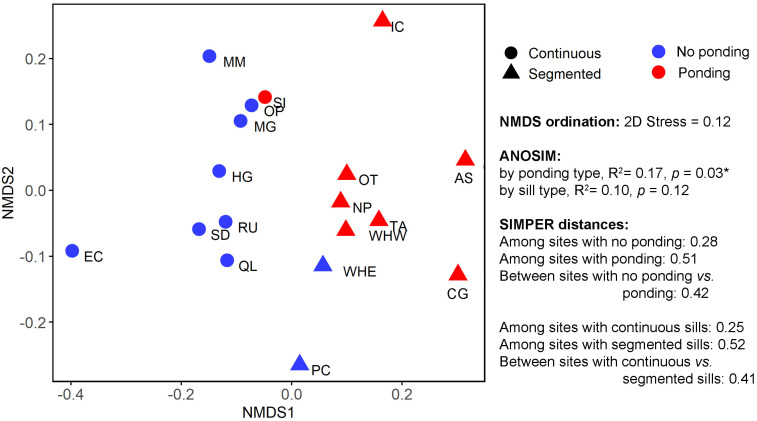
Non-metric multi-dimensional scaling analyses for 18 living shorelines with representative variables including Gap/Rock (G/R) ratio, Elevation relative to Mean Sea Level (m), Sand percentage (%), Relative Exposure Index (REI), Stem density (num/m^2^), and Deposition rate (mm/y). * *p* < 0.05.

The selected six representative metrics were largely independent, except for a moderate correlation between the G/R ratio and Elevation relative to MSL (*r* = 0.610; Table 5), which is unlikely to notably affect the NMDS results. The NMDS analysis yielded a robust ordination of sites, visually indicating clear separation among living shorelines with ponding versus no ponding (Fig 4). An ANOSIM test confirmed that the ponding group was significantly influenced by environmental factors (*p* < 0.05), with the six metrics explaining 17.10% of the observed variability between the ponding versus no ponding group. However, a similar ANOSIM test based on sill types (continuous sill versus segmented sill living shorelines) revealed a relatively low proportion of variance explained (R^2^ = 0.10) by sill type, and sill type did not significantly account for variability in the NMDS data (*p* = 0.12). Notably, Sunset Island (SI) showed little separation from the living shorelines with no ponding, aligning with field observations that ponding was limited to the north in this living shoreline. Given the correlation between G/R ratio and elevation relative to MSL, we further removed one of the two metrics to keep variables independent. Results indicated that removing either of them in the NMDS analysis led to less separation among living shorelines with ponding versus no ponding (S3 Fig). Conversely, Irish Creek (IC), Assateague State Park (AS), and Conrad Gordon (CG) clearly separated from living shorelines with no ponding, reflecting the noticeable presence of open water within these marshes.

The SIMPER analysis revealed significant differences in average squared distances among sites within the ponding versus no ponding groups (0.28 versus 0.51), clearly separating them into two distinct groups on the plot (Fig 4). Similarly, within the sill type comparison (continuous sill versus segmented sill), the average squared distance among sites within the continuous sill group (0.25) was significantly less than within the segmented sill group (0.52), resulting in distinct separation on the plot (Fig 4). The SIMPER analysis identified Relative Exposure Index (REI) and Gap/Rock ratio emerging as significant contributors, explaining 5.46% and 4.41% of the difference between ponding and no ponding groups, respectively (Table 6). Additionally, elevation relative to Mean Sea Level (MSL) and G/R ratio explained 10.42% and 5.72% of the difference between continuous and segmented sill living shorelines, respectively (Table 6).

**Table 6:** Results of SIMPER analyses providing the contribution of each metric to the overall dissimilarity grouped by sill type and ponding type.

Metric	Sill type			Ponding type		
	Contribution (%)	Ratio	*p*-value	Contribution (%)	Ratio	*p*-value
Sand percentage (%)	11.59	1.18	0.70	11.58	1.14	0.70
Elevation relative to Mean Sea Level (m)	10.42	1.39	0.03*	9.89	1.55	0.12
Deposition rate (mm/y)	7.25	1.01	0.73	7.43	0.97	0.46
Stem density (num/m^2^)	7.15	0.91	0.20	6.54	0.90	0.86
Gap/Rock ratio	5.72	0.90	0.01*	5.46	0.82	0.01*
Relative Exposure Index (REI)	4.00	0.58	**0.06.**	4.41	0.61	0.01*

Notes: *p < 0.05;. *p* < 0.1.

## 4. Discussion

UVVR and sediment deposition rate are recognized indicators of tidal marsh resilience [27,47], but their applicability to assessing the stability of marsh sill living shorelines has not been previously explored. In this study, we investigated 18 marsh sill living shorelines in Maryland, comprising 9 with continuous sills and 9 with segmented sills. Our results indicated no significant difference in UVVR and deposition rate between continuous and segmented sill living shorelines. However, living shorelines with ponding at the marsh edge tended to exhibit a significantly higher UVVR (0.14 ± 0.06, large effect size, Cohen’s d = −1.39, *p* < 0.05) and a lower sediment deposition rate (7.14 ± 1.50 mm/y, medium effect size, Cohen’s d = 0.68, *p* < 0.1). Previous research has highlighted that UVVR values greater than 0.1 are indicative of increased open-water conversion and marsh deterioration [27–29]. We identified a significant correlation between UVVR and deposition rate (Fig 2d; R^2^ = 0.19, *p* < 0.1), which was stronger among living shorelines with ponding (S2b Fig; R^2^ = 0.52). These findings align with earlier studies on natural tidal marshes, which emphasized the importance of UVVR and deposition rate as indicators of marsh stability [27,29]. In natural marshes, indicators of marsh stability are often associated with environmental factors that influence marsh dieback, including elevation, sediment supply, and sea-level rise [29]. In this study, we observed a significant positive correlation between sediment deposition rate and Total Suspended Matter (TSM; R^2^ = 0.33, *p* < 0.01), indicating that living shorelines can trap more sediments where coastal nearshore water has higher sediment availability. This finding aligns with earlier research emphasizing the significant role of sediment availability in coastal wetland restoration success [25]. However, not all patterns observed in natural marshes necessarily translate to living shoreline marshes. While previous studies across natural marsh ecosystems have noted a consistent scaling of elevation with UVVR, showing lower elevation units tend to exhibit more open-water conversion and higher UVVRs [28], we found no significant linear correlation between UVVR and elevation relative to Mean Sea Level (MSL). Instead, we identified a significant polynomial relationship between sediment deposition rate and Relative Tidal Marsh Elevation (Z*_MHW_), underscoring the importance of tidal range in living shoreline marshes (Fig 2b). The higher sediment deposition rate in marshes with lower Z*_MHW_ could be attributed to marsh loss and open-water conversion. A tidal marsh that has already experienced vegetation loss may witness increased sediment mobilization through tidal and wave processes, leading to accretion due to longer submergence times [50–52]. Therefore, in marshes with significant vegetation loss, sediment accretion, and subsequent elevation gain may be symptoms of instability rather than drivers of stability [53].

Among the key indicators of marsh stability, functional marsh width (W^*^) is a novel metric that captures the geographic characteristics of fringing marshes. The relationship between W^*^ and sediment deposition rate varied depending on whether the deposition rate exceeded or fell below the relative sea level rise (RSLR). When the deposition rate exceeded the RSLR, a negative scaling relationship is observed (Fig 3b; y = 189.46x^−1.542^, R^2^ = 0.886). Conversely, when the deposition rate was below the RSLR, a positive scaling relationship was evident (Fig 3c; y = 3.473ln(x)−8.275, R^2^ = 0.970). These varying correlations can be explained by the physical processes of sediment transport and deposition. When the deposition rate exceeded the RSLR, the negative scaling relationship suggested diminishing returns as marsh width increased, likely due to reduced sediment concentration or changes in flow dynamics. Initially, wider marshes enhanced sediment deposition by providing more area for sediment to settle and slowing water flow. However, beyond a certain width, the additional benefits decreased. In contrast, when the deposition rate was below the RSLR, the positive scaling relationship indicated that increasing marsh width consistently improved sediment trapping efficiency, though the rate of improvement diminished logarithmically. The relationship reflects how marshes dynamically balance sediment deposition with sea-level rise. Narrow marshes struggle more to keep up with rising seas, but wider marshes may reach a saturation point where additional width decreases efficiency. An optimal marsh width of approximately 5–10 m balanced cost-effectiveness with high deposition rates, effectively leveraging the benefits of increased width without encountering diminishing returns or excessive costs.

Open-water conversion is a primary destructive process in marshes, influencing the long-term fate of marsh complexes [27]. Previous studies have noted that the living shoreline boundary degrades as ponds expand landward of rock sills [23]. We identified this form of ponding at 8 sites, encompassing both continuous and segmented sill living shorelines. Through multivariate analyses, we investigated the main drivers of this ponding process in living shorelines. Results indicated that the Gap/Rock ratio and Relative Exposure Index (REI) made significant contributions to differences among living shorelines with ponding versus no ponding, with contributions of 5.46% and 4.41%, respectively (*p* < 0.05 for both). This suggests that tidal gaps and wind-generated wave energy are primary drivers of ponding in living shorelines. Independent t-tests also showed a significant difference in G/R ratio between living shorelines grouped by ponding and no ponding, with a higher G/R ratio in living shorelines with ponding (0.15 ± 0.08) compared to those without ponding (0.01 ± 0.01; *p* < 0.1), indicating more frequent ponding in segmented sill living shorelines than continuous sill living shorelines. Although REI was dramatically higher in living shorelines with ponding (1011.05 ± 586.16) compared to those without ponding (194.81 ± 26.32), this difference was not statistically significant in the t-test (*p* = 0.2). Among living shorelines grouped by sill types, major contributors to differences between continuous sill and segmented sill living shorelines were relative elevation to Mean Sea Level (MSL) and G/R ratio, with contributions of 10.42% and 5.72%, respectively (*p* < 0.05 for both). This suggests that while consideration was given to marsh elevation or height in living shoreline design, more attention is needed to wave energy to prevent ponding and marsh degradation. While the total variance explained is relatively low, this is not uncommon in field-based ecological and geomorphological studies due to the inherent complexity and variability of natural systems [47,48]. Our goal was to identify broad patterns and key gradients rather than achieve high explanatory power, and the selected metrics still captured meaningful variation across sites.

For marsh managers, the most valuable indicators of marsh resilience in living shorelines should possess predictive power for degradation, enabling optimized design or post-construction maintenance interventions. This predictive capability allows for strategies to prevent marsh degradation through specialized design or timely interventions to restore marshes, such as elevation addition, marsh replanting, sill height adjustment, or facilitating marsh migration to higher ground. Robust predictions often stem from factors directly related to marsh dieback drivers [29]. Some metrics in our analyses signal potential marsh boundary degradation in marsh sill living shorelines. Designers should particularly consider the G/R ratio of gap design in living shorelines. Although tidal gaps are increasingly used to enhance hydrological connectivity and marine fauna access to created marshes, they can also increase tidal water exchange, wave energy transmission, and sediment transportation, impacting erosion/deposition in the created marsh [14]. Notably, living shorelines with a high G/R ratio are likely to develop ponding zones between segmented sills, especially at sites with high wave energy. Designers should account for this, possibly incorporating ponding into the design. For instance, the living shoreline at AS was designed as a headland breakwater with a G/R ratio close to 1, incorporating a ponding area and pocket beach between sill segments into the design.

This study provides valuable insights into optimal living shoreline design and maintenance post-construction, but several important limitations must be acknowledged. One significant limitation is the use of static metrics to depict the current state of living shorelines, rather than tracking changes over time, which may limit the understanding of marsh dynamics and response to environmental stressors. Field sampling conducted across different years within the growing season (2017–2023) introduces temporal variability that could affect data interpretation and trend analysis. Besides, ^7^Be-derived sediment deposition rates are at seasonal scales, while the RSLR is averaged over decades. This discrepancy in the timescale might limit the direct comparability of the two, potentially leading to challenges in accurately correlating sediment dynamics with long-term environmental changes. Additionally, the reliance on Google Earth Pro for satellite imagery-based metrics may introduce uncertainty due to differences in image resolution, data quality, and acquisition methods. Finally, the study’s geographical focus on Maryland”s Chesapeake Bay and Coastal Bays implies that observed patterns and conclusions may not universally apply to other coastal regions with distinct environmental conditions, morphologies, and anthropogenic influences. Despite these limitations, this study provides valuable insights into metrics for assessing the stability of marsh sill living shorelines and major drivers for the potential marsh boundary degradation. The findings contribute to a deeper understanding of the marsh resilience of living shorelines and offer a solid foundation for advancements in shoreline design and post-construction maintenance.

## 5. Conclusions

This study investigated 18 marsh sill living shorelines in Maryland, nine with continuous sills and nine with segmented sills. It assessed the resilience of these marshes under different sediment supply and hydrodynamic conditions to determine if metrics applicable to natural marshes could also predict potential degradation in living shoreline marshes. Utilizing datasets from four prior studies, including 15 metrics obtained through field measurements and remote sensing, we sought to identify common patterns across regional ecosystems. The results suggested that the UVVR and sediment deposition rates effectively differentiated among living shorelines with and without ponding. Moreover, their correlation with other environmental factors like Total Suspended Matter (TSM) and relative tidal marsh elevation (Z*_MHW_) aligned with patterns observed in natural marshes. Among the six representative metrics selected from each category, the Relative Exposure Index (REI) and G/R ratio were identified as significant contributors to the ponding processes at the marsh boundaries (*p* < 0.05). Additionally, we introduced a novel vegetation-related metric, the Functional Marsh Width (W*), to assess the effective width of fringing marshes in living shorelines. Its nonlinear correlation with sediment deposition rates suggested that a width of 5–10 m is optimal for achieving more sediment deposition. Overall, this research not only explores the application of various metrics in assessing marsh resilience but also sheds light on potential causes of boundary degradation in living shorelines, offering possible strategies to optimize marsh-sill complex design and post-construction maintenance.

## Supporting information

S1 Fig Delineation of vegetated and unvegetated areas at living shorelines with various configurations: (a) Continuous sill living shoreline at SI, (b) Segmented sill living shoreline at AS. The green zone represents the vegetated area, and the white zone indicates the unvegetated area. Base aerial images were obtained via drone on April 26, 2022.
(DOCX)

S2 Fig (a) Relationship between the Unvegetated/Vegetated Ratio (UVVR) and Sediment Deposition Rate (mm/y) excluding the outlier site, QL. (b) Relationship between UVVR and Sediment Deposition Rate for sites with UVVR > 0. (c) Relationship between UVVR and Sediment Deposition Rate for sites with segmented sills. Different colors indicate different rates of sea level rise (mm/y).
(DOCX)

S3 Fig (a) Non-metric multi-dimensional scaling analysis for 18 living shorelines with representative variables including Gap/Rock (G/R) ratio, Sand percentage (%), Relative Exposure Index (REI), Stem density (num/m^2^), and Deposition rate (mm/y); (b) Non-metric multi-dimensional scaling analysis for 18 living shorelines with representative variables including Elevation relative to Mean Sea Level (m), Sand percentage (%), Relative Exposure Index (REI), Stem density (num/m^2^), and Deposition rate (mm/y). (DOCX)

S1 Table Summary of Regression Models for Sediment Deposition Rate (mm/y). (DOCX)

S2 Table Dataset used to assess marsh resilience in living shorelines. (DOCX)
