# Supplementary material for: Metrics for assessing stability of marsh sill living shorelines: Identifying main drivers of marsh boundary degradation
Source: PLoS One. 2025 Oct 9;20(10):e0333214. doi: 10.1371/journal.pone.0333214 (PMC12510553; doi:10.1371/journal.pone.0333214)
Supplement: S1 Fig — The green zone represents the vegetated area, and the white zone indicates the unvegetated area. Base aerial images were obtained via drone on April 26, 2022. (DOCX) [file pone.0333214.s001.docx]

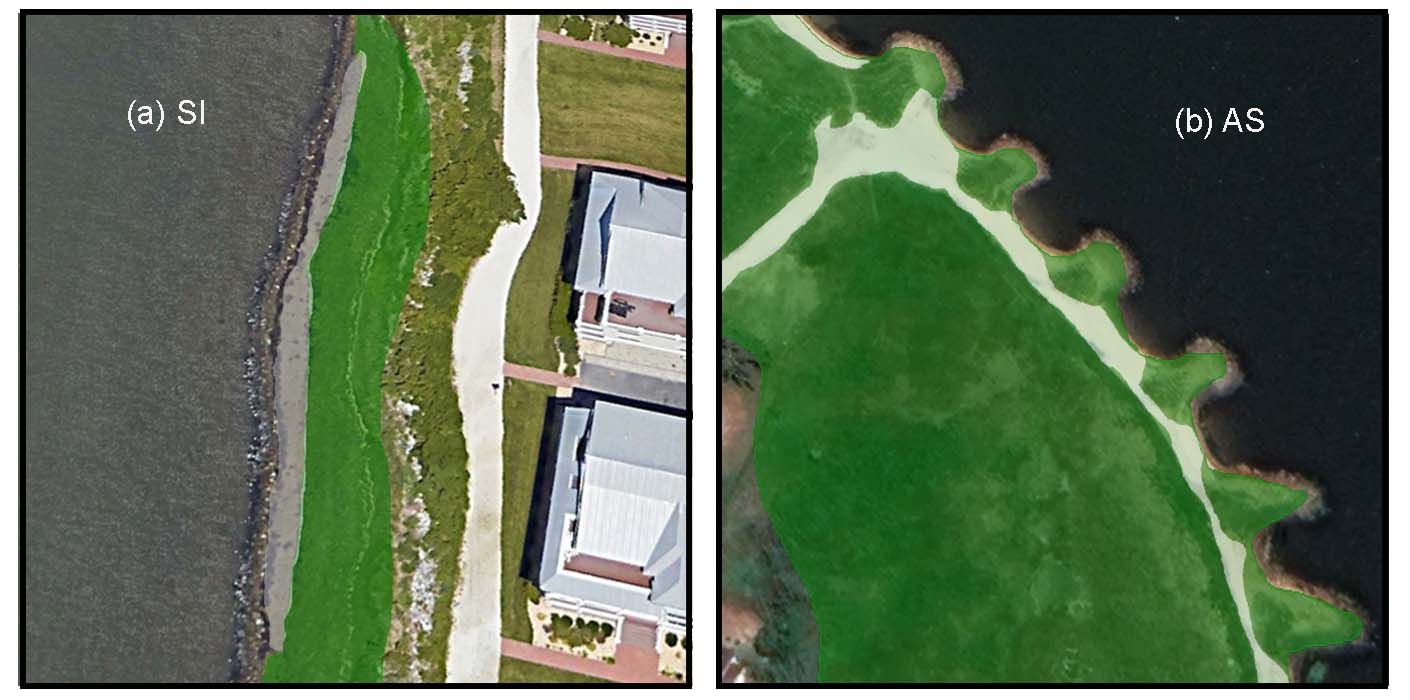


S1 Fig. Delineation of vegetated and unvegetated areas at living shorelines with various configurations: (a) Continuous sill living shoreline at SI, (b) Segmented sill living shoreline at AS. The green zone represents the vegetated area, and the white zone indicates the unvegetated area. Base aerial images were obtained via drone on April 26, 2022.
