# Supplementary material for: Metrics for assessing stability of marsh sill living shorelines: Identifying main drivers of marsh boundary degradation
Source: PLoS One. 2025 Oct 9;20(10):e0333214. doi: 10.1371/journal.pone.0333214 (PMC12510553; doi:10.1371/journal.pone.0333214)
Supplement: S2 Fig — (b) Relationship between UVVR and Sediment Deposition Rate for sites with UVVR > 0. (c) Relationship between UVVR and Sediment Deposition Rate for sites with segmented sills. Different colors indicate different rates of sea level rise (mm/y). (DOCX) [file pone.0333214.s002.docx]

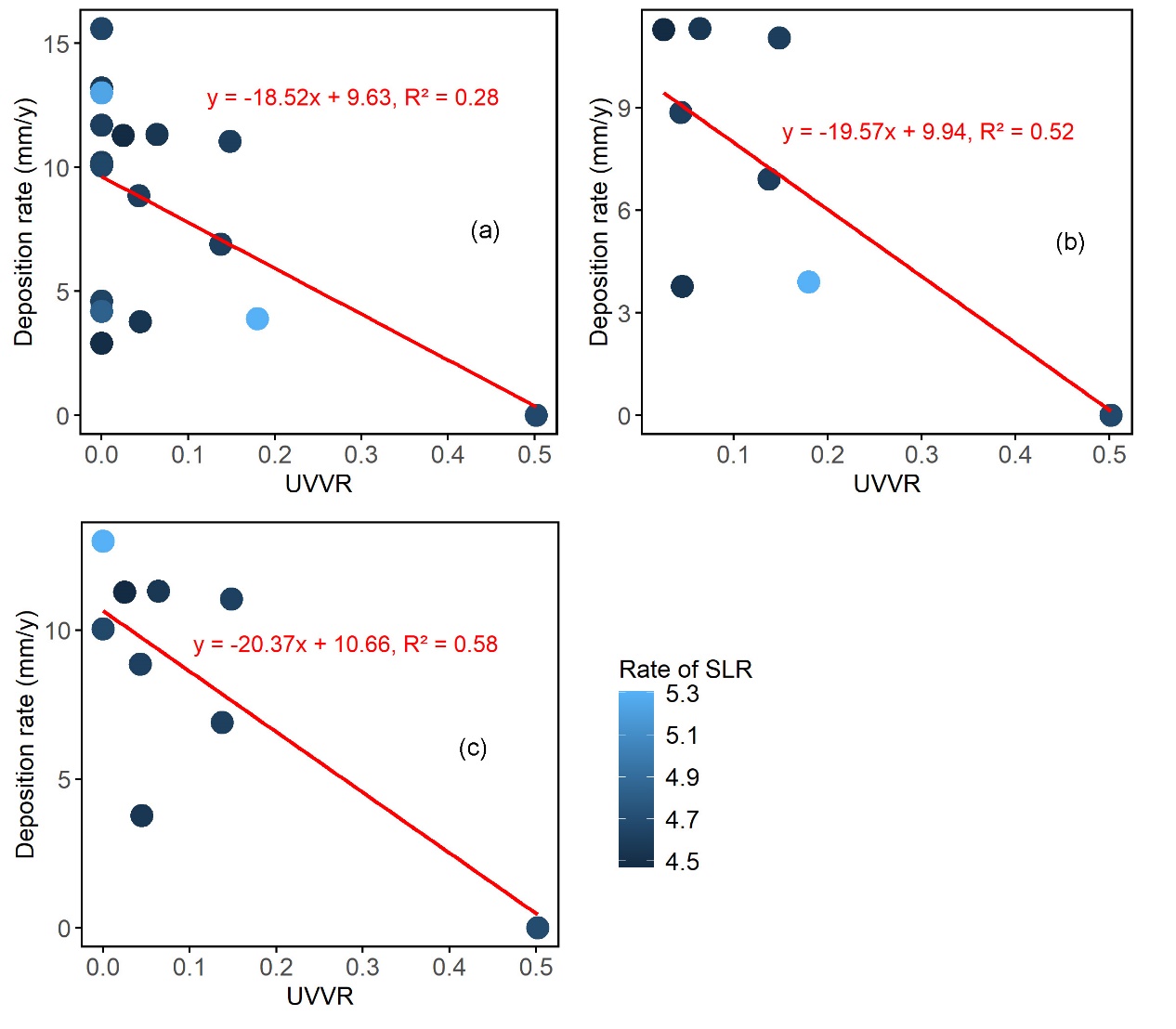


S2 Fig. (a) Relationship between the Unvegetated/Vegetated Ratio (UVVR) and Sediment Deposition Rate (mm/y) excluding the outlier site, QL. (b) Relationship between UVVR and Sediment Deposition Rate for sites with UVVR > 0. (c) Relationship between UVVR and Sediment Deposition Rate for sites with segmented sills. Different colors indicate different rates of sea level rise (mm/y).
