# Supplementary material for: Metrics for assessing stability of marsh sill living shorelines: Identifying main drivers of marsh boundary degradation
Source: PLoS One. 2025 Oct 9;20(10):e0333214. doi: 10.1371/journal.pone.0333214 (PMC12510553; doi:10.1371/journal.pone.0333214)
Supplement: S3 Fig — (DOCX) [file pone.0333214.s003.docx]

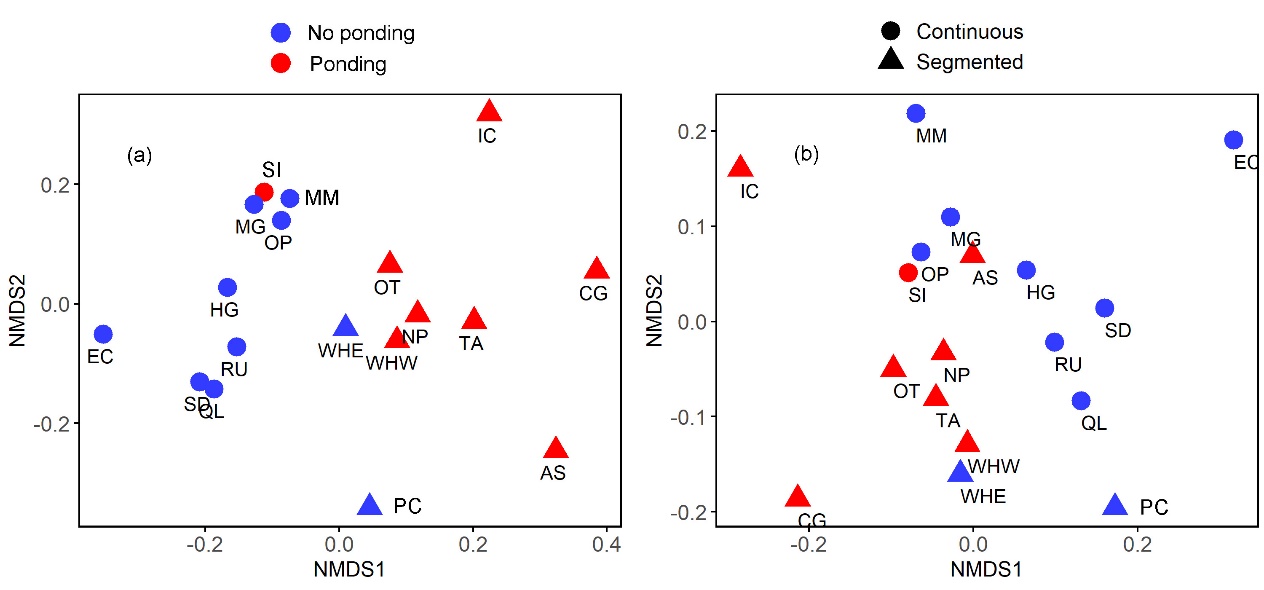


S3 Fig. (a) Non-metric multi-dimensional scaling analysis for 18 living shorelines with representative variables including Gap/Rock (G/R) ratio, Sand percentage (%), Relative Exposure Index (REI), Stem density (num/m^2^), and Deposition rate (mm/y); (b) Non-metric multi-dimensional scaling analysis for 18 living shorelines with representative variables including Elevation relative to Mean Sea Level (m), Sand percentage (%), Relative Exposure Index (REI), Stem density (num/m^2^), and Deposition rate (mm/y).
