# Supplementary material for: Metrics for assessing stability of marsh sill living shorelines: Identifying main drivers of marsh boundary degradation
Source: PLoS One. 2025 Oct 9;20(10):e0333214. doi: 10.1371/journal.pone.0333214 (PMC12510553; doi:10.1371/journal.pone.0333214)
Supplement: S1 Table — (DOCX) [file pone.0333214.s004.docx]

| S4 Table Summary of Regression Models for Sediment Deposition Rate (mm/y). | | | | | | |
| --- | --- | --- | --- | --- | --- | --- |
| Predictor Variable | Equation | Estimate (*β*) | Std. Error | t value | R² | *p* |
| Elevation relative to MSL | *y* = 6.703 + 4.498 *x* | 6.703 | 2.658 | 2.522 | 0.046 | 0.197 |
|  |  | Slope (*x*) | 4.498 | 3.34 |  |  |
| Relative Tidal Marsh Elevation (*Z**_MHW_) | *y* = 8.597 – 2.404 *x* + 0.363 × *x*² | 8.597 | 1.181 | 7.279 | 0.715 | 0.000 |
|  |  | Slope (*x*) | –2.404 | 0.551 |  |  |
|  |  | Slope (*x*²) | 0.363 | 0.06 |  |  |
| Total Suspended Matter (TSM) | *y* = –22.997 + 6.345 *x* | –22.997 | 11.66 | –1.972 | 0.289 | 0.012 |
|  |  | Slope (*x*) | 6.345 | 2.254 |  |  |
| Unvegetated/Vegetated Ratio (UVVR) | *y* = 10.658 – 20.367 *x* | 10.658 | 1.206 | 8.838 | 0.516 | 0.018 |
|  |  | Slope (*x*) | –20.367 | 6.594 |  |  |
| Vegetated Width (Power-law) | *y* = 189.46 *x*^(-1.542) | Intercept: 5.2442 | 0.379 | 13.825 | 0.875 | 0.000 |
|  |  | Slope: -1.5420 | 0.174 | -8.834 |  |  |
| Vegetated Width (Logarithmic) | *y* = 3.474 ln(*x*) - 8.275 | Intercept: -8.2751 | 0.603 | -13.71 | 0.963 | 0.000 |
|  |  | Slope: 3.4735 | 0.303 | 11.45 |  |  |
