# Supplementary material for: Metrics for assessing stability of marsh sill living shorelines: Identifying main drivers of marsh boundary degradation
Source: PLoS One. 2025 Oct 9;20(10):e0333214. doi: 10.1371/journal.pone.0333214 (PMC12510553; doi:10.1371/journal.pone.0333214)
Supplement: S2 Table — (DOCX) [file pone.0333214.s005.docx]

| S5 Table Dataset used to assess marsh resilience in living shorelines | Deposition rate (mm/y) | 0.39 | 3.04 | 0.46 | 1.17 | 1.02 | 1.56 | 1.32 | 0.29 | 0.42 | 0.69 | 1.01 | 1.13 | 1.10 | 0.89 | 1.30 | 0.00 | 1.13 | 0.38 |
| --- | --- | --- | --- | --- | --- | --- | --- | --- | --- | --- | --- | --- | --- | --- | --- | --- | --- | --- | --- |
|  | Unvegetated/vegetated ratio (UVVR) | 0.18 | 0.00 | 0.00 | 0.00 | 0.00 | 0.00 | 0.00 | 0.00 | 0.00 | 0.14 | 0.00 | 0.03 | 0.15 | 0.04 | 0.00 | 0.50 | 0.06 | 0.04 |
|  | Stem density (num/m2) | 116.00 | 189.27 | 106.18 | 109.76 | 138.79 | 122.37 | 412.22 | 132.65 | 118.95 | 281.00 | 95.61 | 112.33 | 123.20 | 189.27 | 11.44 | 81.18 | 165.17 | 46.00 |
|  | Stem height (cm) | 74.80 | 157.33 | 108.99 | 112.73 | 153.32 | 124.00 | 138.73 | 142.36 | 118.52 | 54.60 | 104.98 | 105.27 | 96.82 | 119.44 | 109.83 | 111.30 | 100.13 | 133.60 |
|  | Vegetation coverage (%) | 100.00 | 78.69 | 96.00 | 98.00 | 93.00 | 72.57 | 92.70 | 100.00 | 93.07 | 100.00 | 78.50 | 89.78 | 73.04 | 74.17 | 43.44 | 83.39 | 97.52 | 60.33 |
|  | Rate of relative Sea-level Rise (RSLR, mm/y) | 5.31 | 4.60 | 4.64 | 4.60 | 4.61 | 4.65 | 4.58 | 4.49 | 4.83 | 4.60 | 4.65 | 4.47 | 4.61 | 4.61 | 5.24 | 4.68 | 4.55 | 4.55 |
|  | Average tidal range (m) | 2.17 | 1.31 | 1.44 | 1.48 | 1.48 | 1.47 | 1.47 | 1.60 | 1.47 | 2.21 | 1.54 | 1.52 | 1.56 | 1.37 | 1.55 | 1.51 | 1.46 | 1.60 |
|  | Relative Exposure Index (REI) | 213.95 | 278.83 | 266.17 | 241.20 | 103.03 | 80.18 | 125.44 | 192.38 | 319.94 | 119.45 | 219.57 | 459.53 | 1200.82 | 644.30 | 121.37 | 335.19 | 5016.78 | 98.35 |
|  | Organic matter content (%) | 1.61 | 8.90 | 5.72 | 13.60 | 16.70 | 24.70 | 23.80 | 21.40 | 1.80 | 18.52 | 10.46 | 12.97 | 4.07 | 4.92 | 15.03 | 20.75 | 4.13 | 19.01 |
|  | Sand percentage (%) | 94.62 | 70.00 | 81.00 | 37.30 | 69.20 | 45.40 | 38.10 | 49.60 | 97.80 | 82.20 | 75.10 | 68.96 | 88.05 | 75.77 | 61.30 | 31.38 | 70.57 | 55.61 |
|  | Total Sediment Matter (TSM, mg/l) | 5.27 | 6.61 | 4.94 | 4.89 | 4.97 | 5.00 | 4.64 | 5.12 | 4.92 | 4.80 | 4.02 | 5.47 | 6.25 | 5.69 | 5.60 | 4.57 | 5.02 | 4.74 |
|  | Relative tidal marsh elevation (Z*MHW, m) | 1.22 | 11.06 | 2.20 | -0.87 | -0.77 | -1.36 | 1.57 | 5.04 | 1.12 | 2.90 | 6.31 | 7.89 | -2.03 | 4.47 | 9.48 | 4.06 | 7.38 | 3.00 |
|  | Elevation relative to Mean Sea Level (m) | 0.59 | 1.14 | 0.30 | 0.41 | 0.35 | 0.30 | 0.08 | 0.28 | 0.11 | 0.73 | 1.75 | 1.47 | 0.88 | 1.24 | 0.43 | 0.27 | 0.69 | 0.63 |
|  | Gap/Rock (G/R) ratio | 0 | 0 | 0 | 0 | 0 | 0 | 0 | 0 | 0 | 0.68 | 0.02 | 0.07 | 0.21 | 0.10 | 0.10 | 0.03 | 0.11 | 0.03 |
|  | Functional marsh width (W*, m) | 7.78 | 4.91 | 11.39 | 8.53 | 9.85 | 6.69 | 7.17 | 7.09 | 8.57 | 20.14 | 9.69 | 7.44 | 8.80 | 11.35 | 6.78 | 2.87 | 8.00 | 7.56 |
|  | pond | y | n | n | n | n | n | n | n | n | y | n | y | y | y | n | y | y | y |
|  | type | Continuous | Continuous | Continuous | Continuous | Continuous | Continuous | Continuous | Continuous | Continuous | Segmented | Segmented | Segmented | Segmented | Segmented | Segmented | Segmented | Segmented | Segmented |
|  | Site | Sunset island | Queens Landing | Oppenheim | Ruesch | Hatton Garden | San Domingo | Environmental Concern | Myrtle Grove | CB Maritime Museum | Assateague State Park | Wye House East | Wye House West | Oxford Beach | Narrows Pointe | Plaindealing Creek | Irish Creek | Conrad Gordon | Old Trinity Church |
